# Expanding radiotherapy access in Sub-Saharan Africa: an analysis of travel burdens and patient-related benefits of hypofractionation

**DOI:** 10.3389/fonc.2023.1136357

**Published:** 2023-04-18

**Authors:** Saloni Patel, Elizabeth Olatunji, Abba Mallum, Binsila Bernard Benjika, Adedayo O. Joseph, Shomari Joseph, Nwamaka Lasebikan, Habiba Mahuna, Mamsau Ngoma, Twalib Athumani Ngoma, Godwin Nnko, Chinelo Onwualu, Mariza Vorster, Wilfred Ngwa

**Affiliations:** ^1^ Johns Hopkins University School of Medicine, Baltimore, MD, United States; ^2^ Department of Radiotherapy and Oncology, University of KwaZulu-Natal, Durban, South Africa; ^3^ Department of Oncology, Inkosi Albert Luthuli Central Hospital, Durban, South Africa; ^4^ Department of Public Health, ICT University USA, Yaoundé, Cameroon; ^5^ NSIA-LUTH Cancer Center, Lagos University Teaching Hospital, Lagos, Nigeria; ^6^ Ocean Road Cancer Institute, Dar Es Salaam, Tanzania; ^7^ Oncology Center, University of Nigeria Teaching Hospital, Ituku Ozalla, Enugu, Nigeria; ^8^ Department of Clinical Oncology, Muhimbili University of Health and Allied Sciences, Dar Es Salaam, Tanzania; ^9^ College of Health Science, University of KwaZulu-Natal, Durban, South Africa; ^10^ Brigham and Women’s Hospital, Dana-Farber Cancer Institute, Harvard Medical School, Boston, MA, United States

**Keywords:** hypofractionated radiotherapy, sub-Saharan Africa, prostate cancer, breast cancer, travel distance, cost savings, time savings

## Abstract

**Purpose:**

The purpose of this project was to examine the travel burdens for radiotherapy patients in Nigeria, Tanzania, and South Africa, and to assess the patient-related benefits of hypofractionated radiotherapy (HFRT) for breast and prostate cancer patients in these countries. The outcomes can inform the implementation of the recent Lancet Oncology Commission recommendations on increasing the adoption of HFRT in Sub-Saharan Africa (SSA) to enhance radiotherapy access in the region.

**Methods:**

Data were extracted from electronic patient records at the NSIA-LUTH Cancer Center (NLCC) in Lagos, Nigeria and the Inkosi Albert Luthuli Central Hospital (IALCH) in Durban, South Africa, from written records at the University of Nigeria Teaching Hospital (UNTH) Oncology Center in Enugu, Nigeria, and from phone interviews at Ocean Road Cancer Institute (ORCI) in Dar Es Salaam, Tanzania. Google Maps was used to calculate the shortest driving distance between a patient’s home address and their respective radiotherapy center. QGIS was used to map the straight-line distances to each center. Descriptive statistics were used to compare transportation costs, time expenditures, and lost wages when using HFRT versus conventionally fractionated radiotherapy (CFRT) for breast and prostate cancer.

**Results:**

Patients in Nigeria (n=390) traveled a median distance of 23.1 km to NLCC and 86.7 km to UNTH, patients in Tanzania (n=23) traveled a median distance of 537.0 km to ORCI, and patients in South Africa (n=412) traveled a median distance of 18.0 km to IALCH. Estimated transportation cost savings for breast cancer patients in Lagos and Enugu were 12,895 Naira and 7,369 Naira, respectively and for prostate cancer patients were 25,329 and 14,276 Naira, respectively. Prostate cancer patients in Tanzania saved a median of 137,765 Shillings in transportation costs and 80.0 hours (includes travel, treatment, and wait times). Mean transportation cost savings for patients in South Africa were 4,777 Rand for breast cancer and 9,486 Rand for prostate cancer.

**Conclusion:**

Cancer patients in SSA travel considerable distances to access radiotherapy services. HFRT decreases patient-related costs and time expenditures, which may increase radiotherapy access and alleviate the growing burden of cancer in the region.

## 1 Introduction

Sub-Saharan Africa (SSA) is facing a growing cancer crisis. A recent Lancet Oncology Commission predicts that, if current trends continue, overall cancer deaths in SSA could increase to about 1 million deaths per year by 2030 (1). Cancers of the breast and prostate are the most common cancers among women and men, respectively, in SSA (2). It is estimated that in the year 2020, 206,710 people in SSA were diagnosed with either breast or prostate cancer and another 104,285 died from their disease (2). Despite the region’s projected increase in cancer incidence and mortality, access to radiotherapy services remains unacceptably low. There is a paucity of radiotherapy machines in SSA, limited trained radiation oncology staff, and long patient travel distances to radiotherapy centers (1). The majority of SSA countries have less than 1 radiotherapy machine per 1 million people, in stark contrast to high-income countries, which have 5 or more machines per 1 million people (3).

This inadequate access is complicated by the fact that external beam radiotherapy (EBRT) is the most appropriate treatment for men with intermediate-and high-risk prostate cancer and is considered the standard of care for breast cancer patients following breast-conserving surgery (4, 5). Hypofractionated radiotherapy (HFRT) is a form of EBRT that provides a potential solution to low radiotherapy access in resource-limited settings. HFRT increases the dose of radiation administered per treatment fraction, reducing the overall length of treatment. Randomized phase III trials have supported the clinical efficacy of HFRT for breast and prostate cancer when compared to conventionally fractionated radiotherapy (CFRT) (6–16). Additionally, the reduction in overall treatment duration and increases in national cost savings may improve the treatment capacity of radiotherapy centers, yielding increased access to RT (17). The Lancet Oncology Commission for SSA recommends increased adoption of HFRT to augment such access (1).

While the potential financial advantages of HFRT for African hospitals and governments have been reported (17), there is a paucity of research conducted on the patient-related benefits of adopting HFRT in SSA. Historically, compared to HFRT, conventional fractionation schedules are more time-consuming for patients. Conventional radiotherapy typically involves 25 fractions administered over 5 weeks for breast cancer (25-30 fractions in 5-6 weeks if including a boost) and 35 to 40 fractions over 7 to 8 weeks for prostate cancer. HFRT reduces the number of visits to the radiotherapy center to approximately 15 and 20 visits for breast and prostate cancer, respectively (10, 15). The longer treatment regimens associated with CFRT can increase financial hardship and pose an inconvenience for patients in SSA, leading to treatment nonadherence or abandonment. Through this study, we sought to close the literature gap and explore the benefits of HFRT from a patient-centered perspective. First, we examined the travel distances of cancer patients from their home address to their respective radiotherapy center in Nigeria, Tanzania, and South Africa. Second, we assessed the patient-related benefits of HFRT for breast and prostate cancer patients in these three countries.

## 2 Materials and methods

Following IRB approvals, primary data collection occurred across four sites between June – November 2022: two in Nigeria, one in Tanzania, and one in South Africa. Patient addresses were extracted from electronic records at the NSIA-LUTH Cancer Center (NLCC) in Lagos, Nigeria and from written records at the University of Nigeria Teaching Hospital (UNTH) Oncology Center in Enugu, Nigeria. Included patients from NLCC were all breast and prostate cancer patients who started receiving either HFRT or CFRT at the NLCC between February 1- July 27, 2022. Included patients from UNTH Oncology Center were all breast or prostate cancer patients who started receiving either HFRT or CFRT at the center between July 1, 2021 – August 4, 2022.

Patient addresses and transportation cost data were extracted from electronic records at the Inkosi Albert Luthuli Central Hospital (IALCH) in Durban, South Africa. Included patients from IALCH were all breast and prostate cancer patients who started receiving HFRT at the center between December 1, 2021 – June 29, 2022.

Phone interviews were conducted in June 2022 among prostate cancer patients who had received or were receiving HFRT at Ocean Road Cancer Institute (ORCI) in Dar es Salaam, Tanzania between January 6 – June 16, 2022. Interviews were conducted in Swahili and responses were translated to English. Phone interviews rather than electronic medical records were used to obtain data from ORCI due to the increased feasibility of gathering additional data on time expenditures and wage savings through direct patient interviews. Google Maps was used to calculate the shortest driving distance between a patient’s home address and their respective radiotherapy center. QGIS was used to map the straight-line distances to each center. Data collected from all four sites were used to analyze transportation cost savings. Transportation cost for a single fraction comprised of the transportation cost to and from the patient’s respective radiotherapy center. Transportation costs for patients in Nigeria were estimated based on the round-trip bus fare between a patient’s accommodation in Lagos or Enugu and NLCC or UNTH (1,271 Naira and 706 Naira, respectively, as of July 2022). This assumption was made based on the fact that most patients remained in either Lagos or Enugu for the duration of their treatment period. Data collected from ORCI were also used to analyze time expenditure savings and wage savings. Time expenditure for a single fraction comprised of the sum of the transportation time to and from ORCI, the patient’s waiting time at the radiotherapy center, and the treatment time for one fraction of HFRT. Descriptive statistics were used to compare transportation costs, time expenditures, and lost wages when using HFRT versus CFRT for breast and prostate cancer. Statistics were calculated based solely on patients with complete data. All patient-related cost and time savings were estimated on the basis of what the transportation costs, time expenditures, and lost wages would have been had the patients received CFRT (defined as 25 visits for breast cancer and 40 visits for prostate cancer) instead of HFRT (15 visits for breast cancer and 20 visits for prostate cancer). Calculations were based on a CFRT regimen of 25 visits for breast cancer and 40 visits for prostate cancer, and a HFRT regimen of 15 visits for breast cancer and 20 visits for prostate cancer since these are the fractions that were implemented across the cancer centers in this study. Additionally, while some studies have established the clinical efficacy of as few as 5 fractions for breast cancer (16), these shorter treatment regimens require investments in high technology equipment which is not feasible in many SSA countries, and calculations using these fractions may not be as generalizable to the region as a whole.

All transportation- and wage-related cost data were contextualized within each country’s monthly adjusted net national income (MANNI) per capita, as reported by 2020 data from the World Bank. These values are 143 USD, 76 USD, and 387 USD for Nigeria, Tanzania, and South Africa, respectively.

## 3 Results

### 3.1 Patient characteristics

**Table 1** displays the characteristics of the interviewed HFRT prostate cancer patients at ORCI. Of the 30 patients who were eligible for interview, data were collected from 23 patients. 4 patients were unable to be contacted, 2 patients had not yet started treatment, and 1 patient elected to opt out of treatment.

**Table 1:** Characteristics of interviewed patients (n=23) at Ocean Road Cancer Institute (ORCI) in Dar Es Salaam, Tanzania.

Parameter	Value	
Median age (range)	68 years	(58-79)
Age (%)		
50-59 years	2	9%
60-69 years	11	48%
70-79 years	10	43%
Prostate Cancer Stage		
II	12	52%
III	7	31%
IV	4	17%
Outpatient v. Inpatient		
Outpatient	22	96%
Inpatient	1	4%
Marital Status		
Married	21	91%
Widowed	2	9%
Religion		
Christian	14	61%
Muslim	9	39%
Education Level		
Primary	7	30%
Secondary	9	40%
College/University	7	30%
Retired (%)	9	39%
Working (%)	14	61%

The mean age of breast cancer patients at NLCC, UNTH, and IALCH was 48, 46, and 59 years respectively, and the mean age of prostate cancer patients was 67, 68, and 70 years, respectively.

### 3.2 Travel distances

**Figure 1:**
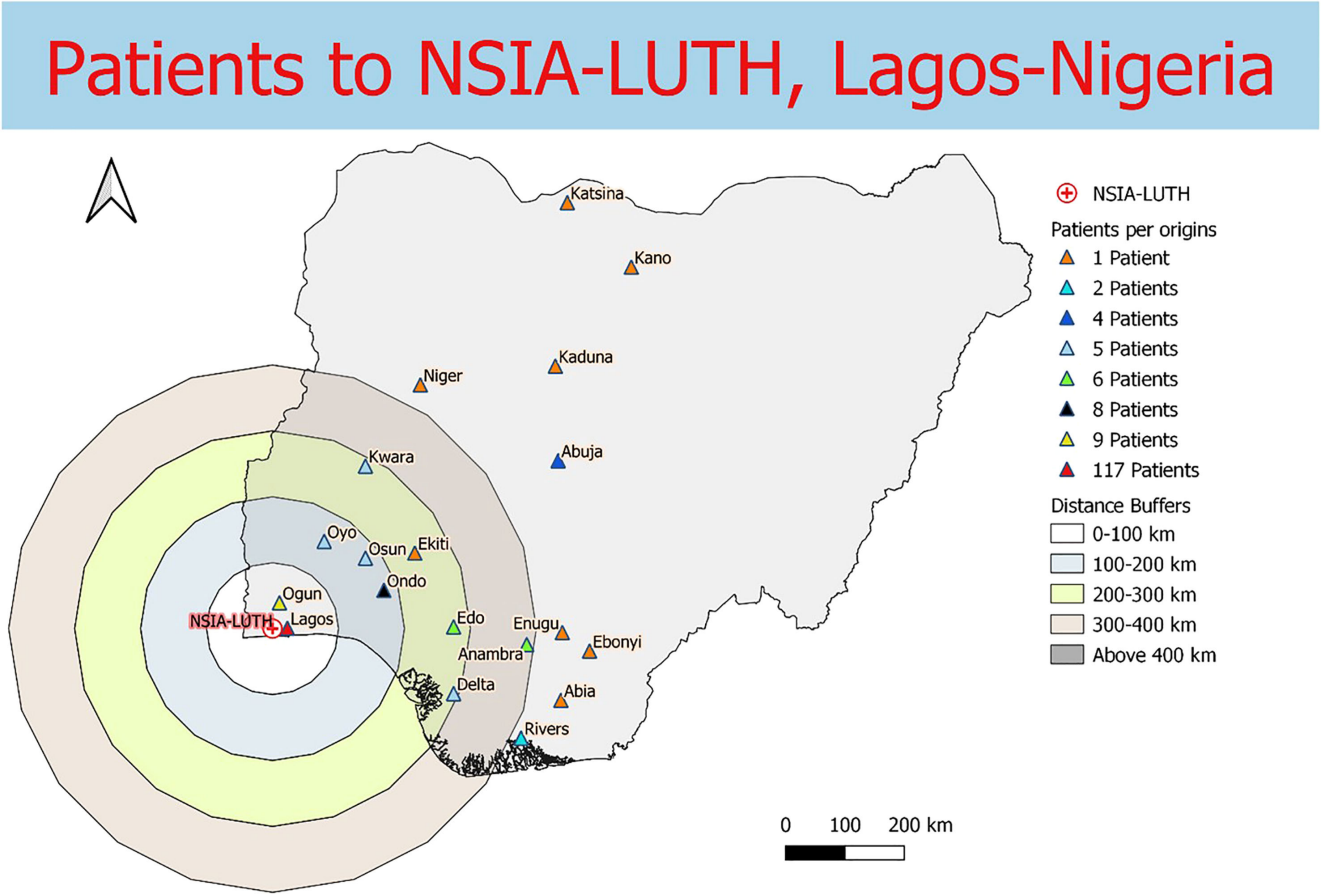
Straight-line distances between the home addresses of radiotherapy patients (n=180) and NSIA-LUTH Cancer Center in Lagos, Nigeria.

**Figure 2:**
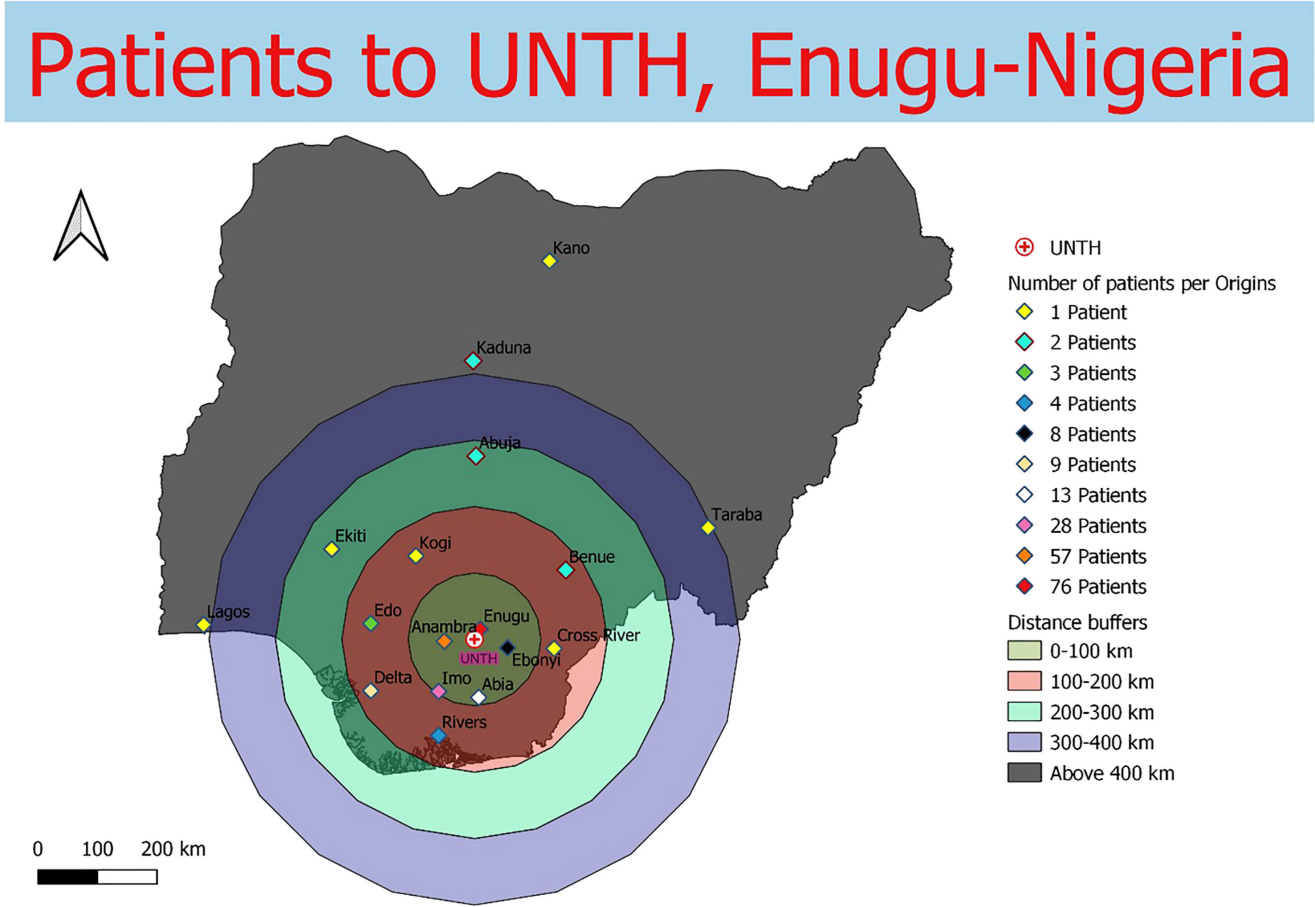
Straight-line distances between the home addresses of radiotherapy patients (n=211) and UNTH Oncology Center in Enugu, Nigeria.

**Figure 3:**
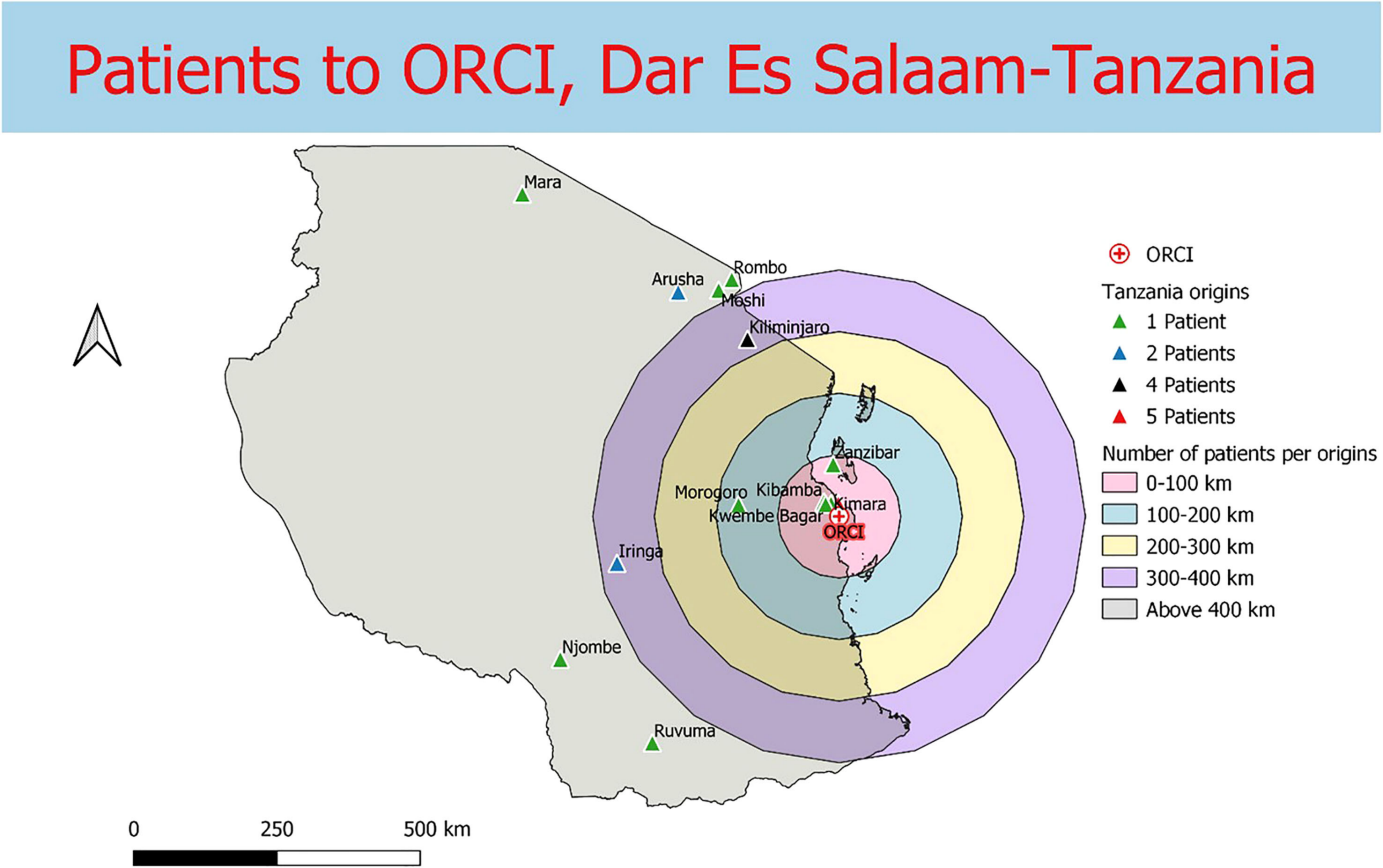
Straight-line distances between the home addresses of radiotherapy patients (n=23) and Ocean Road Cancer Institute (ORCI) in Dar Es Salaam, Tanzania.

**Figure 4:**
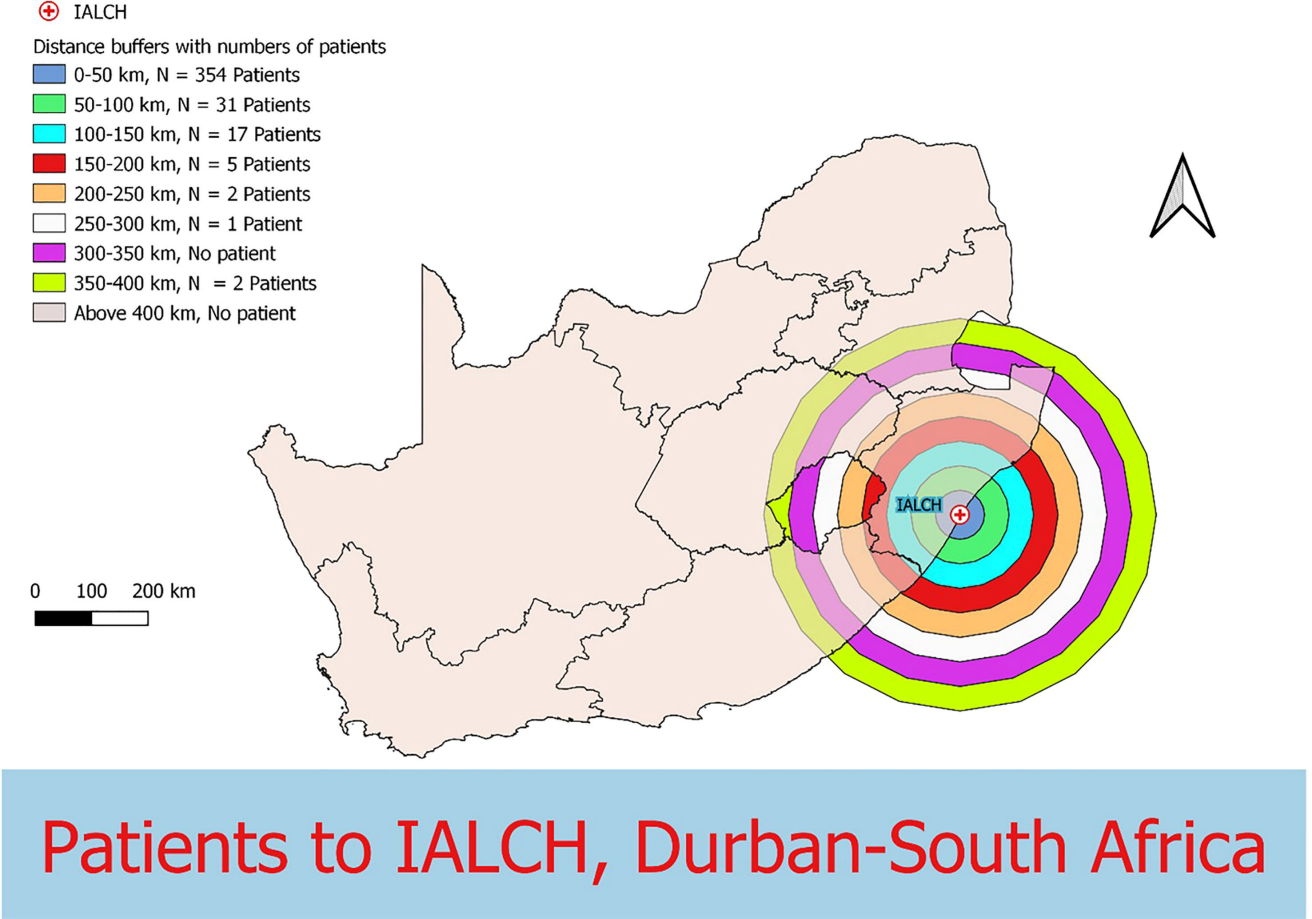
Straight-line distances between the home addresses of radiotherapy patients (n=412) and Inkosi Albert Luthuli Central Hospital (IALCH) in Durban, South Africa.

Data were collected from 180 breast and prostate cancer patients who traveled to NLCC, 211 breast and prostate cancer patients who traveled to UNTH, 23 prostate cancer patients who traveled to ORCI, and 412 breast and prostate cancer patients who traveled to IALCH. Patients in Nigeria traveled a median distance of 23.1 km (IQR=109.2 km) and 86.7 km (IQR=87.3 km) to NLCC and UNTH, respectively (**Figures 1**, **2**). Patients in Tanzania traveled a median of 537.0 km (IQR=587.5 km) to ORCI (**Figure 3**). Patients in South Africa traveled a median of 18.0 km (IQR=15.0 km) to IALCH (**Figure 4**). These findings are summarized in **Table 2**. The QGIS maps in **Figures 1–4** were made using straight-line distances rather than the road-network distances that were calculated with Google Maps. Therefore, these figures depict shorter-than-actual travel distances.

**Table 2:** Median travel distances of patients to four different radiotherapy centers across Sub-Saharan Africa.

Radiotherapy Center	Median Travel Distance (km)	First Quartile (km)	Third Quartile (km)	Interquartile range (km)
Ocean Road Cancer Institute (ORCI) Dar Es Salaam, Tanzania	537.0	27.0	614.5	587.5
NSIA-LUTH Cancer Center (NLCC) Lagos, Nigeria	23.1	12.8	122	109.2
University of Nigeria Teaching Hospital (UNTH) Enugu, Nigeria	86.7	28.2	115.5	87.3
Inkosi Albert Luthuli Central Hospital (IALCH) Durban, South Africa	18.0	13.0	28.0	15.0

### 3.3 Transportation cost and time expenditure savings

**Figure 5:**
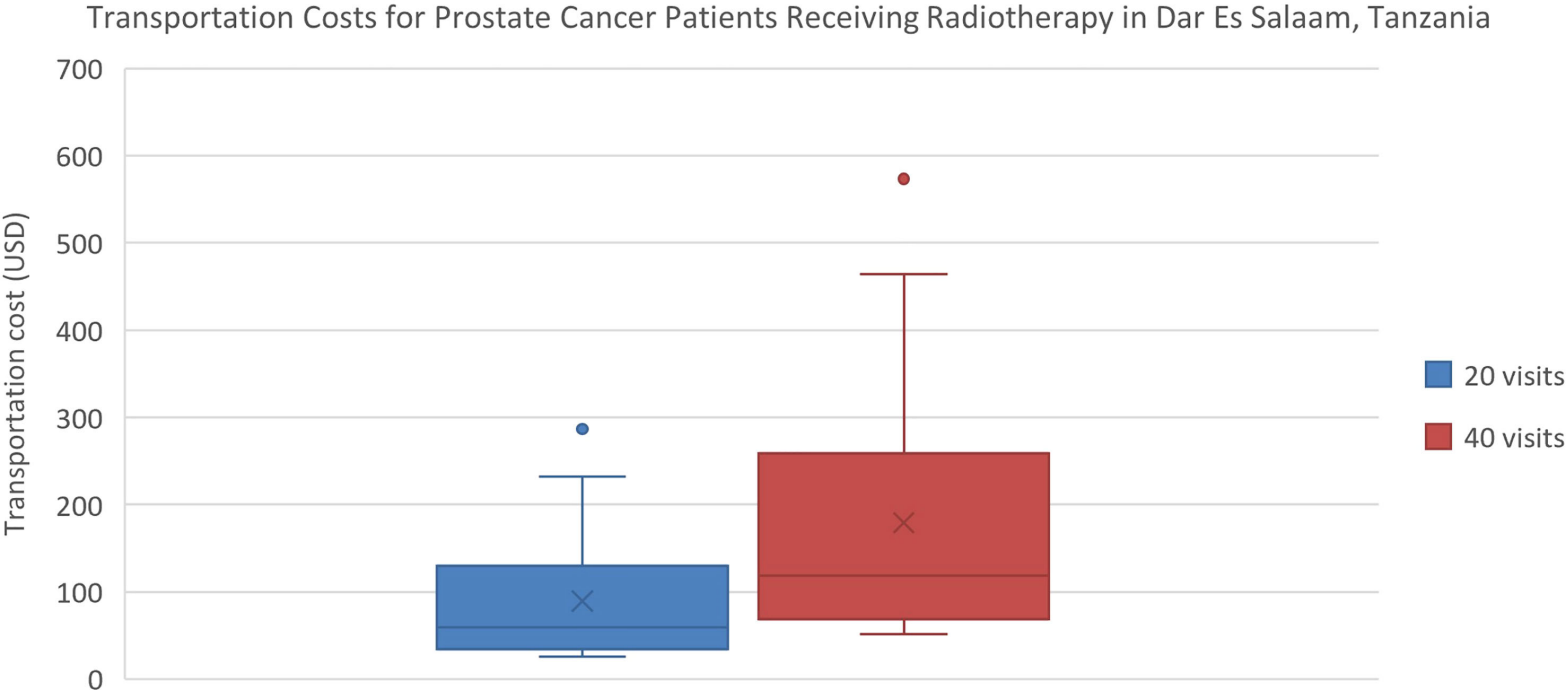
Comparison of transportation costs for prostate cancer patients at ORCI in Dar Es Salaam, Tanzania. 20 visits represents the HFRT course, and 40 visits represents the CFRT course. Error bars depict the interquartile range.

**Figure 6:**
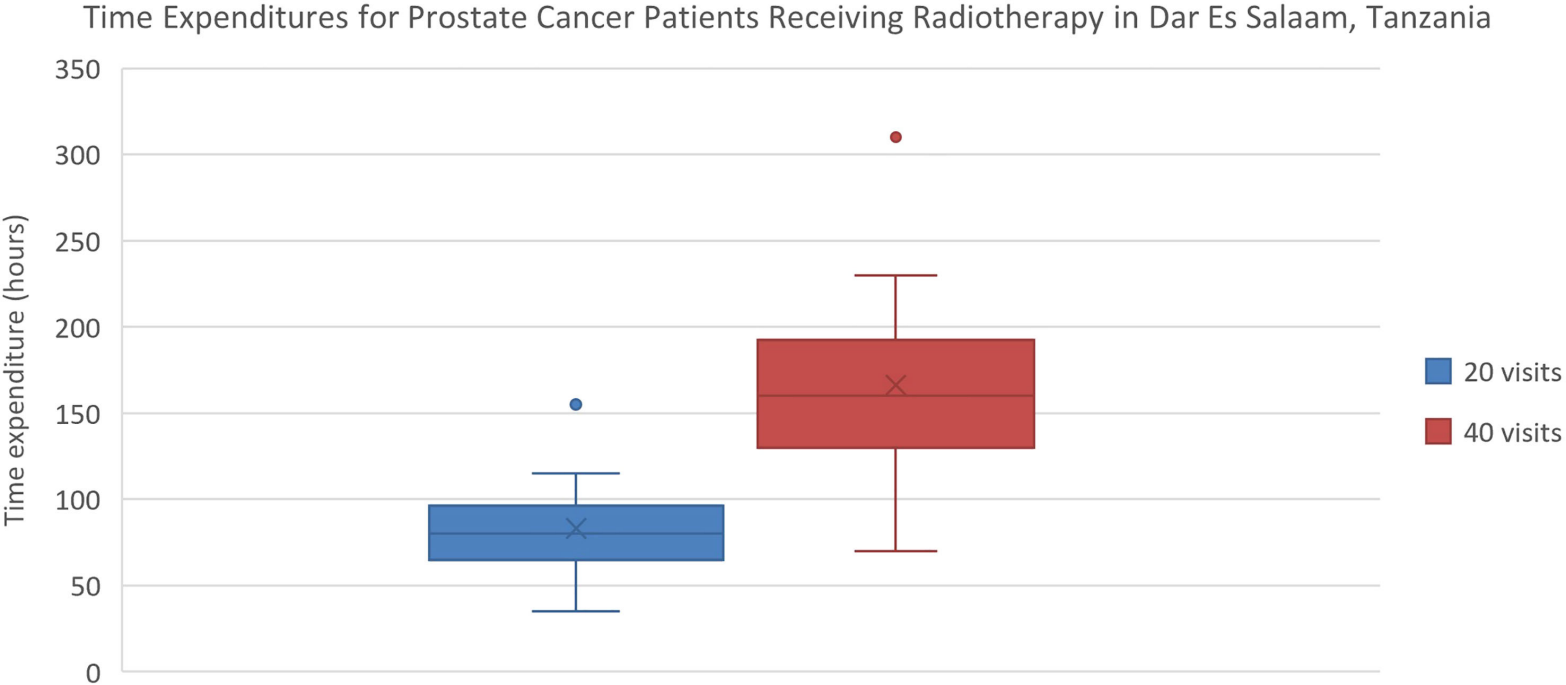
Comparison of time expenditures for prostate cancer patients at ORCI in Dar Es Salaam, Tanzania. 20 visits represents the HFRT course, and 40 visits represents the CFRT course. Error bars depict the interquartile range.

Estimated transportation cost savings for breast cancer patients in Lagos and Enugu were 12,895 Naira (20% of MANNI per capita) and 7,368 Naira (11% of MANNI per capita), respectively and for prostate cancer patients were 25,329 (38% of MANNI per capita) and 14,276 Naira (22% of MANNI per capita), respectively (**Tables 3**, **4**). 22of the 23 ORCI respondents were outpatients, and data from these 22 prostate cancer patients were used to analyze transportation cost and time expenditure savings. Adopting hypofractionation saved ORCI patients a median of 137,765 shillings (78% of MANNI per capita, IQR= 203,145 shillings) in transportation costs and 80.0 hours (IQR=30.0 hours) over the course of their radiotherapy treatment (**Figures 5**, **6**). Mean transportation cost savings for IALCH patients were 4,771 rand for breast cancer (72% of MANNI per capita) and 9,474 rand for prostate cancer (143% of MANNI per capita) (**Table 5**).

**Table 3:** Comparison of estimated transportation costs for breast and prostate cancer patients at NSIA-LUTH Cancer Center (NLCC) in Lagos, Nigeria. Hypofractionation is 15 visits for breast cancer and 20 visits for prostate cancer.

	Conventional fractionation (Naira)	Hypofractionation (Naira)	Estimated Transportation Cost Savings (Naira; % of monthly adjusted net national income per capita)
Breast Cancer	31,777	18,882	12,895 (20%)
Prostate Cancer	50,659	25,330	25,329 (38%)

Conventional fractionation is 25 visits for breast cancer and 40 visits for prostate cancer. Costs are given in the Nigerian currency Naira.

**Table 4:** Comparison of estimated transportation costs for breast and prostate cancer patients at University of Nigeria Teaching Hospital (UNTH) Oncology Center in Enugu, Nigeria.

	Conventional fractionation (Naira)	Hypofractionation (Naira)	Estimated Transportation Cost Savings (Naira; % of monthly adjusted net national income per capita)
Breast Cancer	17,961	10,592	7,369 (11%)
Prostate Cancer	28,553	14,277	14,276 (22%)

Hypofractionation is 15 visits for breast cancer and 20 visits for prostate cancer. Conventional fractionation is 25 visits for breast cancer and 40 visits for prostate cancer. Costs are given in the Nigerian currency Naira.

**Table 5:** Comparison of transportation costs for breast and prostate cancer patients at Inkosi Albert Luthuli Central Hospital (IALCH) in Durban, South Africa.

	Conventional fractionation (Rand)	Hypofractionation (Rand)	Estimated Transportation Cost Savings (Rand; % of monthly adjusted net national income per capita)
Breast Cancer	11,936	7,165	4,771 (72%)
Prostate Cancer	18,948	9,474	9,474 (143%)

Hypofractionation is 15 visits for breast cancer and 20 visits for prostate cancer. Conventional fractionation is 25 visits for breast cancer and 40 visits for prostate cancer. Costs are given in the South African currency Rand.

### 3.4 Wage savings

All non-retired ORCI patients (61%) reported temporary absenteeism from work to receive treatment. Of these, 12 patients were wage-earning, and 75% of wage-earning patients (largely peasant farmers and business owners) did not receive sick leave compensation and incurred lost wages due to treatment. By undergoing HFRT instead of CFRT, these patients saved a median of 100,405 shillings (57% of monthly NNI per capita, IQR= 51,370 shillings) in lost wages.

### 3.5 Reported barriers and challenges

43% of ORCI patients reported challenges getting to the clinic for their daily treatment fractions. The most common barrier cited was a lack of empty seating on public transportation buses and resultant delays in getting to the radiotherapy center. Additionally, 61% of patients reported experiencing a negative financial impact due to the aforementioned patient-related expenses incurred during their treatment course.

## 4 Discussion

Adopting HFRT instead of CFRT in SSA reduces the transportation costs, time expenditures, and lost wages incurred by patients. When contextualized within the monthly adjusted net national income (MANNI) per capita for each country, the transportation cost and wage-related savings represent sizable portions of a typical patient’s monthly income and are significant economic savings from a patient-centered perspective. Since approximately 40% of SSA’s population lives below the poverty line, transportation and wage-related cost savings are important considerations for expanding RT access to the region’s most vulnerable communities (18). Additionally, many of the ORCI patients, particularly those who were not working prior to their course of HFRT, relied on their family members for financial support to offset costs associated with their treatment. Therefore, the financial impact of cancer treatment is felt by many individuals beyond the patient, and cost savings associated with HFRT benefit family units as a whole.

While salary data was solely collected from ORCI patients in Tanzania, the GDP per capita can be used to calculate a rough estimation of the wage savings for HFRT patients in Nigeria and South Africa. According to 2021 data from the World Bank, the GDP per capita for Nigeria and South Africa is 173.75 USD/month and 582.85 USD/month, respectively (19). Assuming that patients report temporary absenteeism from work for the duration of their treatment and do not receive sick leave compensation, estimated wage savings for breast cancer patients are 86.88 USD in Nigeria and 291.43 USD in South Africa, and for prostate cancer patients are 173.75 USD in Nigeria and 582.85 USD in South Africa.

Furthermore, our project illustrates the far distances that many patients have to travel to access radiotherapy treatment in SSA due to the scarce distribution of treatment centers in the region. Compared to patients in Tanzania, the calculated travel distances for patients in Nigeria and South Africa are shorter, likely due to the larger sample sizes and the increased number of radiotherapy centers in these two countries. However, the shorter travel distances for patients in Nigeria and South Africa do not capture the full travel burden associated with attending radiotherapy treatment, as traffic in large SSA cities, such as Lagos, can extend travel times for patients tremendously (20, 21). Potential transportation time savings associated with traffic congestion are reflected in our results, which, along with our findings on reductions in clinic wait times, are especially relevant for increasing patient convenience and treatment adherence in resource-limited settings. Additionally, the large IQR values obtained for NLCC and ORCI in particular (238.5 km and 587.5 km, respectively) reveal the wide range of distances that patients are travelling to obtain radiotherapy.

Inadequate access to radiotherapy machines has been cited as a major contributing factor to the disproportionately high – and increasing – cancer mortality rates in SSA (22). While all three countries fall short of the recommendations set forth by the IAEA of one radiation therapy unit per 200,000 persons, patients in South Africa have significantly better access to RT compared to patients in Nigeria and Tanzania (23, 24). South Africa has 1 RT machine per 608,000 persons, which is far more than Nigeria’s 1 machine per 29.4 million persons and Tanzania’s 1 machine per 11.9 million persons (24). These disparities in RT access are reflected in our findings, as patients in South Africa travelled the shortest median distance to their radiotherapy center.

Despite the cost and convenience benefits associated with adoption of HFRT, many of the interviewed patients reported facing barriers in getting to the clinic due to public transportation challenges and experiencing economic hardship as a result of patient-related treatment expenses. These challenges would have been exacerbated if the patients had adopted a conventionally fractionated regimen due to the additional visits to the radiotherapy center. Documentation of these challenges provides an incentive for exploration of treatments such as ultrahypofractionated radiation therapy in SSA to further reduce the number of visits to the radiotherapy center and improve existing patient burdens (25).

There are some limitations to this study. First, the small sample size of patients interviewed at ORCI limited the amount of data that could be extracted to calculate patient-related benefits. ORCI began offering HFRT for prostate cancer in January 2022, and when the interviews were conducted in June 2022, only 30 patients had met eligibility requirements for this study. Thus, fewer patients could be recruited from ORCI compared to the other centers. Additionally, the patient-related costs analyzed in this study do not include the cost of radiotherapy fractions. There are some patient-related costs, such as food costs and accommodation costs that were unaccounted for. This likely means that total patient-related expenses for the treatment course were underestimated. However, the vast majority of interviewed patients stayed with their family member(s) while in Dar es Salaam, so accommodation expenses were less relevant for this study. The calculated transportation cost savings for patients in Nigeria were estimated with the assumption that patients used a public transport bus to travel to and from the radiotherapy center each day. However, it is possible that these values could be over or under estimated if patients used a personal vehicle for transportation instead. Furthermore, our analyses did not address all aspects of the patient-related benefits of HFRT, such as potential psychosocial benefits. Also, as our project was limited to three countries across SSA, further research in this area could benefit from involving additional SSA countries and could be extended within the context of other common cancers within the region, such as cervical cancer. Lastly, the different methodologies used to collect data in the three countries does limit the extent to which the results may be generalizable. While transportation cost data was acquired directly from patient interviews in Tanzania, they were acquired more indirectly in South Africa from electronic medical records and were estimated in Nigeria. These differing methodologies highlight the lack of a cohesive medical record system throughout SSA, however, the necessary approximations in this study were reasonably calculated within an Afro-centric context. Additionally, while travel distances data was collected from both CFRT and HFRT patients in Nigeria and South Africa, data was collected solely from HFRT patients in Tanzania, potentially limiting the generalizability of these results to radiotherapy patients as a whole in SSA. However, it is worth noting that countries within SSA are diverse in transportation and medical infrastructure and while our analysis attempts to capture this significant regional variability in radiotherapy patient travel burden, it is not all-encompassing.

Despite these limitations, our project provides valuable analyses and perspectives on travel distances and the patient-related benefits of HFRT over CFRT for radiotherapy patients in SSA. Factors such as travel burdens, transportation costs, and lost wages are important barriers to consider for all patients seeking radiotherapy and are especially pertinent for patients in low- and middle-income countries. HFRT is better equipped to address these challenges in a patient-centered manner and its widespread adoption holds potential to alleviate the increasing cancer burden in SSA as recommended by the recent Lancet Oncology Commission (1).

## Data availability statement

The raw data supporting the conclusions of this article will be made available by the authors, without undue reservation.

## Ethics statement

The studies involving human participants were reviewed and approved by Office of Research Integrity University of Massachusetts Lowell IRB#: 22-087. The patients/participants provided their written informed consent to participate in this study.

## Author contributions

Conceptualization and design: SP, EO, AM, MV, WN. Project administration and supervision: AOJ, NL, MN, TAN, CO, MV, WN. Investigation: SP, EO, SJ, HM, GN, AM, MV. Data interpretation: SP, EO, BBB. Writing-original draft: SP. Writing-reviewing and editing: all authors. All authors contributed to the article and approved the submitted version.
